# Stable Structure and Fast Ion Diffusion: A Flexible MoO_2_@Carbon Hollow Nanofiber Film as a Binder-Free Anode for Sodium-Ion Batteries with Superior Kinetics and Excellent Rate Capability

**DOI:** 10.3390/polym16111452

**Published:** 2024-05-21

**Authors:** Na Feng, Mingzhen Gao, Junyu Zhong, Chuantao Gu, Yuanming Zhang, Bing Liu

**Affiliations:** 1 State Key Laboratory of Bio-Fibers and Eco-Textiles, Qingdao University, No. 308 Ningxia Road, Qingdao 266071, China; 2 College of Materials Science and Engineering, Qingdao University, No. 308 Ningxia Road, Qingdao 266071, China; 3 College of Textiles & Clothing, Qingdao University, No. 308 Ningxia Road, Qingdao 266071, China; 4 School of Environmental and Municipal Engineering, Qingdao University of Technology, No. 777 Jialingjiang East Road, Qingdao 266520, China

**Keywords:** hollow structure, sodium-ion battery, ion diffusion

## Abstract

Designing innovative anode materials that exhibit excellent ion diffusion kinetics, enhanced structural stability, and superior electrical conductivity is imperative for advancing the rapid charge–discharge performance and widespread application of sodium-ion batteries. Hollow-structured materials have received significant attention in electrode design due to their rapid ion diffusion kinetics. Building upon this, we present a high-performance, free-standing MoO_2_@hollow carbon nanofiber (MoO_2_@HCNF) electrode, fabricated through facile coaxial electrospinning and subsequent heat treatment. In comparison to MoO_2_@carbon nanofibers (MoO_2_@CNFs), the MoO_2_@HCNF electrode demonstrates superior rate capability, attributed to its larger specific surface area, its higher pseudocapacitance contribution, and the enhanced diffusion kinetics of sodium ions. The discharge capacities of the MoO_2_@HCNF (MoO_2_@CNF) electrode at current densities of 0.1, 0.2, 0.5, 1.0, 2.0 and 5.0 A g^−1^ are 195.55 (155.49), 180.98 (135.20), 163.81 (109.71), 144.05 (90.46), 121.16 (71.21) and 88.90 (44.68) mAh g^−1^, respectively. Additionally, the diffusion coefficients of sodium ions in the MoO_2_@HCNFs are 8.74 × 10^−12^ to 1.37 × 10^−12^ cm^2^ s^−1^, which surpass those of the MoO_2_@CNFs (6.49 × 10^−12^ to 9.30 × 10^−13^ cm^2^ s^−1^) during the discharging process. In addition, these prepared electrode materials exhibit outstanding flexibility, which is crucial to the power storage industry and smart wearable devices.

## 1. Introduction

The market demand for both power and energy storage batteries has been increasing year after year due to the ongoing growth of the new energy vehicle and energy storage markets, and battery systems have promising growth prospects. However, the development of lithium-ion batteries (LIBs) may be restricted due to the limitations of the raw material reserves [1]. Sodium-ion batteries (SIBs) possess lower material costs, with an abundance of metallic sodium reserves, and have broad research and application prospects [2,3,4]. Meanwhile, SIBs possess a smaller working voltage window than LIBs, making the electrolyte difficult to break down, improving safety and opening up a wide range of research and application possibilities [5]. However, there are several challenges in the study of SIBs. The ionic radius of Na^+^ (0.113 nm) is around 35% larger than that of Li^+^ (0.076 nm), resulting in embedding that is difficult to reverse and slow kinetics. The volume change in SIBs is more violent than that of LIBs during the electrochemical reaction, and the slow diffusion kinetics of sodium ions hinders the promotion of its practical application [6,7]. Therefore, it is essential to strengthen the kinetic study of the sodium ion diffusion process.

Ion diffusion kinetics is the basis for storing charges in electrochemical energy storage devices, which is crucial for battery electrochemical reactions and significantly impacts the performance of electrode materials. The main methods to improve the ion diffusion kinetics of electrode materials include morphology control, the use of composites, nanosize applications, etc. [8,9]. Among these methods, morphology control can improve the ion diffusion rate by changing the morphology of the material, such as spherical, rod-like, flake-like, tubular, etc., which can affect the diffusion path and direction of ions [10,11]. In addition, the hollow structure with internal voids not only provides more active sites but also increases the contact area between the electrode and the electrolyte, thus increasing the reaction rate and capacity of the electrode. Meanwhile, it can shorten the ions’ diffusion path and reduce the diffusion resistance, thus improving the ion diffusion rate and the rate performance of the electrode. Furthermore, the hollow structure of the electrode material can greatly relieve the dramatic volume change during electrochemical reactions, which also can strengthen the structural stability of the electrode and cycle life [12,13,14]. Therefore, the construction of hollow-structured anode materials provides an effective strategy for the pursuit of higher performance in energy storage batteries. Zou et al. developed a novel anode material that was prepared for application in LIBs and SIBs. This Ni-MOFs with layered hollow sphere-in-sphere nanostructures had a capacity of 207 mAh g^−1^ at a current density of 2 A g^−1^, with a specific capacity attenuation of 0.2% cycles per revolution [15]. Uniform nitrogen–carbon doping on bullet-shaped Cu_9_S_5_ hollow particles was performed by Fang et al., exhibiting excellent multiplicity performance and a long cycle life [14]. All of these works encourage the construction of hollow nanostructures to improve ion diffusion kinetics as electrodes for SIBs [16].

At present, the anode materials used for SIBs are mostly carbon-based anode materials (including graphite, amorphous carbon, nanocarbon, etc.), alloy anode materials, metal oxide and sulfur-based anode materials, embedded titanium anode materials, and organic anode materials [17,18]. Among them, carbon-based anode materials have good electrical conductivity but low embedded sodium capacity and poor kinetics [19,20]. Alloy anode materials exhibit high theoretical capacity, but the more drastic volume change leads to lower coulomb efficiency, worse cycling stability, and inferior energy density. Metal oxide anode materials have high theoretical capacity but poor electrical conductivity [9,21]. Embedded titanium anode materials have a small volume change but poor capacity. Organic anode materials have low cost, but poor conductivity and are easily soluble in electrolytes [22,23]. Therefore, it is useful to design metal oxide composite carbon anode materials, which have high specific capacity, high electrical conductivity, and excellent cycling stability. Among many anode materials, molybdenum dioxide (MoO_2_) is considered a very attractive anode material for batteries, with a vast interlayer distance and high theoretical capacity [24,25]. However, its low conductivity and sluggish diffusion kinetics seriously hinder its application, and most of the current work on MoO_2_-based anode materials in SIBs focuses on exploring the enhancement of their electrochemical performance without a more in-depth study of their ion diffusion kinetics (Table S1, Supporting Information) [6,26]. Hence, to reveal the intrinsic mechanism of hollow-structured MoO_2_-based anode materials for enhancing the performance of SIBs, the present work validates the kinetics of the sodium ion diffusion reaction from different perspectives and defines the modulation of the hollow structure as a general strategy.

Herein, we thoroughly investigate the kinetic behavior of the sodium ion diffusion reaction of hollow-structured anode materials compared to solid-structured anode materials in SIBs to design superior anode materials that can greatly improve the electrochemical performance of SIBs. In this work, we constructed a hollow carbon nanofiber film uniformly loaded with MoO_2_ via coaxial electrospinning and subsequent heat treatment, which was pressed to directly obtain electrode sheets. This method is not only simple and facile to operate, but also eliminates the need for additives and binders, thus reducing the occurrence of side reactions. Additionally, the experimental results verify that the obtained MoO_2_@HCNF electrode has a larger contact reaction area, a shorter ion diffusion path, more active sites, excellent multiplicity performance, and faster ion diffusion kinetics in sodium-ion batteries compared to those of the MoO_2_@CNF electrode. Therefore, it is feasible to promote charge transfer and enhance the ion diffusion kinetics by constructing hollow structures.

## 2. Materials and Methods

### 2.1. Materials 

Polyacrylonitrile (PAN, Mw = 150,000) and polymethylmethacrylate (PMMA, Mw = 35,000) were purchased from Shanghai Macklin Biochemical Co., Ltd., Shanghai, China. Ammonium molybdate tetrahydrate (AMMT) and N, N-dimethylformamide (DMF) were supplied by Sinopharm Chemical Reagent Co., Ltd., Shanghai, China.

### 2.2. Synthesis of MoO_2_@HCNFs and MoO_2_@CNFs

Firstly, 1.2 g of PAN was dispersed in 8.8 g of DMF and stirred for 4 h at 40 °C to form a pale yellow clarified solution. Subsequently, 0.72 g of AMMT was added and stirred at room temperature overnight, and the final solution formed was named the shell layer solution. Next, 3 g of PMMA was dispersed in 7 g of DMF and stirred overnight at room temperature, and the final solution obtained was named as the core layer solution. Subsequently, the shell layer solution and the core layer solution were transferred into 5 mL syringes and connected to a 17/22-gauge needle for coaxial spinning to obtain the nanofibrous membrane. The working voltage, air humidity, tip-to-collector distance, flow rate of the shell solution, and flow rate of the core solution were fixed at 20 kV, 40%, 18 cm, 0.4 mL h^−1^, and 0.2 mL h^−1^, respectively. The nanofibrous membrane was pre-oxidized in a muffle furnace at 270 °C for 2 h at a heating rate of 2 °C min^−1^ and then moved to a tube furnace and carbonized at 600 °C for 2 h at a heating rate of 3 °C min^−1^ under nitrogen atmosphere to obtain the MoO_2_@HCNFs. As a comparison, the MoO_2_@CNFs were synthesized in the same way as MoO_2_@HCNFs without the core layer solution.

### 2.3. Material Characterization

Powder X-ray diffraction (XRD) patterns were recorded on a Rigaku Ultima IV diffractometer (Tokyo, Japan) with Cu Kα (λ = 0.154184 nm) radiation at 40 kV and 40 mA. Field emission scanning electron microscopy (FE-SEM) images were collected on the JEOL JSM-7800F microscope (Tokyo, Japan). High-resolution transmission electron microscope (HRTEM) images and energy dispersive spectroscopy (EDS) mapping were investigated via a JEOL JEM-F200 Plus microscope (Tokyo, Japan). X-ray photoelectron spectroscopy (XPS) spectra were obtained using a ULVAC PHI 5000 Versa Probe Ispectrometer (Chigasaki, Japan) with a monochromatized Al Ka X-ray source (1486.6 eV). Raman spectra were studied using a Horiba LabRAM HR Evolution microscope (Kyoto, Japan) with a wavelength of 532 nm. Thermogravimetric analysis (TGA) was carried out to calculate the active material contents of the samples using METTLER TOLEDO (Zurich, Switzerland) at the heating rate of 10 °C min^−1^ under an air atmosphere. Nitrogen adsorption–desorption isotherms were examined on a Quantachrome Autosorb-iQ3 (Boynton Beach, FL, USA).

### 2.4. Electrochemical Measurements

The electrochemical tests were performed using coin-type cells (CR2025), which were assembled in an Ar-filled glove box (H_2_O < 0.1 ppm, O_2_ < 0.1 ppm). The as-prepared flexible MoO_2_@HCNFs and MoO_2_@CNFs were dried at 80 °C in a vacuum, cut into a disc with a diameter of 14 mm, and used as the cathode electrode directly without any additives. A Na metal foil was used as the counter electrode. The glass microfiber filter (Whatman, grade GF/A) served as the separator. The electrolyte was 1.0 M sodium perchlorate (NaClO_4_) in ethylene carbonate (EC)/diethyl carbonate (DEC) (1:1 by volume) with 5 wt % fluoroethylene carbonate (FEC). Galvanostatic charge–discharge (GCD) and galvanostatic intermittent titration technique (GITT) tests were tested on the NEWARE battery testing system at room temperature. Cyclic voltammetry (CV) and electrochemical impedance spectroscopy (EIS) tests were performed on an electrochemical workstation (CHI 660E, Shanghai Chenhua, Shanghai, China). The CV curves were performed in the voltage range from 0.01 to 3.00 V (vs. Na/Na^+^) with different scanning rates. The EIS was measured in the frequency range of 100 kHz–0.01 Hz.

## 3. Results and Discussion

**Figure 1:**
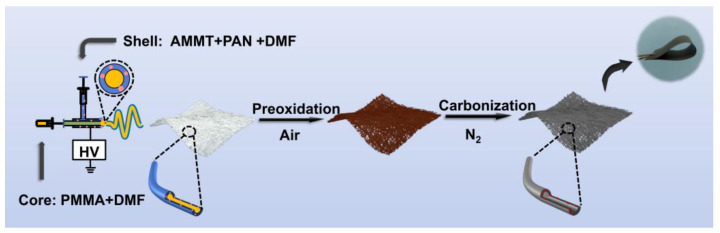
Schematic illustration of the synthetic process of MoO_2_@HCNF composites via the electrospinning method.

The synthesis process of MoO_2_@hollow carbon nanofibers (MoO_2_@HCNFs) is shown in Figure 1. Firstly, we formed a shell-core fiber structure through a simple coaxial electrospinning method, after pre-oxidation and carbonization under a nitrogen atmosphere at 600 °C. The ammonium molybdate in the shell layer thermally decomposed to form MoO_2_, while the PMMA in the core layer completely decomposed to form the hollow carbon nanofiber structure. This method of synthesizing carbon nanofibers with a hollow structure is straightforward to operate, and the obtained hollow structure is uniform and smooth, which promotes shortening the transport channel of electrons and ions, accompanied by alleviating volume expansion. Meanwhile, the synthesized carbon nanofiber film can be directly laminated into an electrode sheet without the need for either a conductive agent and binder or an aluminum foil as a collector, reducing the occurrence of side reactions as well as lowering the cost.

**Figure 2:**
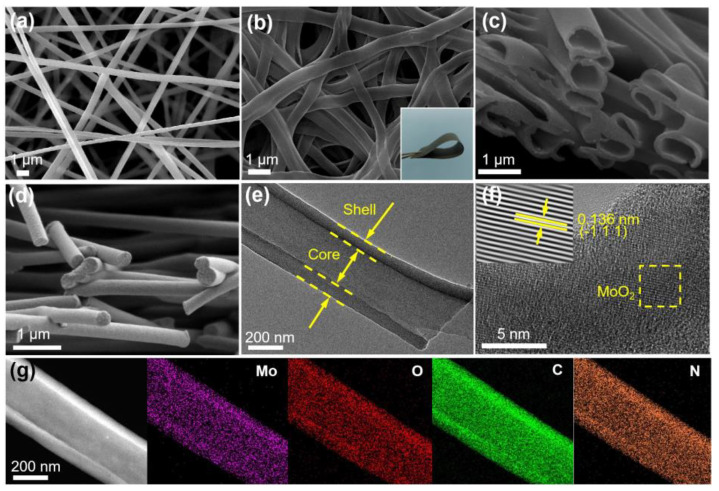
(**a**−**c**) SEM images of the MoO_2_@HCNFs (inserted: photograph of flexible MoO_2_@HCNFs film); (**d**) SEM image of the MoO_2_@CNFs; (**e**) FETEM image of the MoO_2_@HCNFs; (**f**) HRTEM image of the MoO_2_@HCNFs; (**g**) high−angle annular dark−field (HAADF) image and corresponding elemental mapping images of the MoO_2_@HCNFs.

Field emission scanning electron microscopy (FE-SEM) and high-resolution transmission electron microscopy (HRTEM) were used to observe the morphologies of the MoO_2_@HCNFs and MoO_2_@carbon nanofibers (MoO_2_@CNFs). The fibers of the MoO_2_@HCNFs both before (Figure 2a) and after (Figure 2b,c) carbonation were very smooth, with regular and hollow shapes of the fibers. The whole fibrous membranes were mutually supportive and developed a three-dimensional conductive network, similar to that of MoO_2_@CNFs. In addition, the prepared electrode material exhibits excellent foldability that does not break after 180° bending, which indicates superior flexibility properties (Figure S1, Supporting Information). To distinguish the hollow features between MoO_2_@HCNFs and MoO_2_@CNFs, the cross-sectional images are shown in Figure 2c,d. Moreover, the TEM image of MoO_2_@HCNFs also shows the hollow features of the fibers in more detail in Figure 2e. The MoO_2_@HCNFs exhibit a hollow feature with thick walls on both sides and thin in the middle, whereas the MoO_2_@CNFs exhibit a uniform solid feature. The fiber diameters of both the MoO_2_@HCNFs and the MoO_2_@CNFs are about 400 nm. The thickness of the hollow carbon wall is around 65 nm. The HRTEM image in Figure 2f demonstrates lattice fringes of 0.136 nm, corresponding to the (−111) plane of the MoO_2_ (JCPDS: 32-0671), which is consistent with the 26.03° diffraction peak in X-ray diffraction (XRD). Figure 2g shows the element distribution of the MoO_2_@HCNFs using element mapping technology, exhibiting obvious shell and core layers. The fibers have a clear hollow structure with Mo, O, C, and N elements concentrating on both sides of the fibers, which indicates that MoO_2_ is dispersed uniformly in the walls of the hollow carbon fibers without agglomeration. The carbon materials can strengthen the electrical conduction of electrode materials, and can also limit the expansion of MoO_2_ to avoid crystal pulverization and electrode damage caused by excessive stress [27].

**Figure 3:**
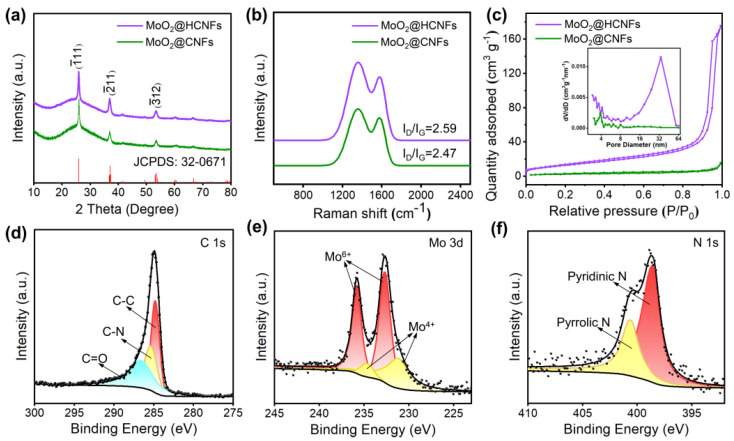
(**a**) XRD patterns of the MoO_2_@HCNFs and MoO_2_@CNFs; (**b**) Raman spectra of the MoO_2_@HCNFs and MoO_2_@CNFs; (**c**) nitrogen adsorption−desorption isotherm curves of the MoO_2_@HCNFs and MoO_2_@CNFs (inserted: pore size distribution curves); high−resolution XPS spectra of the MoO_2_@HCNFs: (**d**) C 1s, (**e**) Mo 3d, and (**f**) N 1s.

The crystal structure and components of the MoO_2_@HCNFs and MoO_2_@CNFs were characterized by means of XRD. These diffraction peaks are located at 26.03°, 37.02°, and 53.51°, which are indexed to the (−111), (−211) and (−312) crystal planes of MoO_2_ (JCPDS: 32-0671), respectively (Figure 3a) [28]. Furthermore, in Figure 3b, the Raman spectra of the MoO_2_@HCNFs and MoO_2_@CNFs exhibit two obvious bands located at 1358 cm^−1^ and 1582 cm^−1^, which are attributed to the D-band and G-band, respectively [29,30]. The calculated I_D_/I_G_ ratio of the MoO_2_@HCNFs is 2.59, which is higher than that of the MoO_2_@CNFs (2.47), suggesting that more active sites and abundant defects were formed in the hollow structure. Furthermore, both the specific surface area and pore size greatly affect the electrochemical properties of electrode materials, and thus the nitrogen adsorption–desorption isotherms and pore size distribution curves of the MoO_2_@HCNFs and MoO_2_@CNFs are shown in Figure 3c. Both the MoO_2_@HCNFs and MoO_2_@CNFs exhibit reversible type IV isotherms, which are mesoporous materials. According to the Brunauer–Emmett–Teller (BET) model, the specific surface area of MoO_2_@HCNFs (46.98 m^2^ g^−1^) is significantly higher than that of the MoO_2_@CNFs (7.87 m^2^ g^−1^), which demonstrates that the electrode material has a larger reaction contact area with the electrolyte in MoO_2_@HCNFs. Both MoO_2_@HCNFs and MoO_2_@CNFs have abundant mesoporous structures, and the pore size of MoO_2_@HCNFs is concentrated at around 32 nm, which is larger than that of MoO_2_@CNFs at around 4 nm, which may be more favorable for the rapid diffusion of ions and accelerate the electrochemical reaction kinetics. Therefore, a uniformly loaded MoO_2_ carbon nanofiber film with simple synthesis and excellent hollow structure was successfully prepared and directly pressed as an electrode sheet without the need for conductive agents and additives in this work. Moreover, according to the thermal gravimetric analysis (TGA, Figure S2, Supporting Information), the MoO_2_@HCNF and MoO_2_@CNF film started to lose weight rapidly after 350 °C, corresponding to the oxidation of carbon in the samples. Meanwhile, the residual MoO_3_ may be generated by the oxidation of MoO_2_ with weight retention of 37.9% and 40.9%, respectively. Based on these data, the MoO_2_ content of the MoO_2_@HCNFs and MoO_2_@CNFs was calculated to be 33.69% and 36.35%, respectively [28,31].

The surface elemental chemical states of electrode materials were tested by means of X-ray photoelectron spectroscopy (XPS). The full survey spectrum (Figure S3, Supporting Information) indicates that the MoO_2_@HCNFs are composed of Mo, C, N, and O elements. The peaks of the C 1s (Figure 3d) are divided into three peaks located at 284.80 eV, 285.36 eV, and 286.63 eV, assigned to C-C, C-N, and C=O bonds, respectively [28,32,33]. The peaks of Mo 3d could be divided to four components (Figure 3e), including two peaks located at 231.19 eV and 234.66 eV of Mo^4+^ and two peaks located at 232.64 eV and 235.81 eV of Mo^6+^ [28,31]. The presence of Mo^6+^ could be assigned to the oxidation of MoO_2_. The signal of N 1s could be clearly distinguished as two peaks with binding energies of 398.55 eV and 400.60 eV (Figure 3f), corresponding to pyridinic nitrogen and pyrrolic nitrogen, respectively, further confirming the presence of nitrogen. The spectrum of O 1s in Figure S3b corresponds to the lattice oxygen of MoO_2_ at the peak at 531.89 eV and shows oxygen-containing functional groups at 533.00 eV [30,34]. Meanwhile, nitrogen-doped carbon materials can introduce multiple defects, which correspondingly result in more active reaction sites and also improve electrical conductivity.

**Figure 4:**
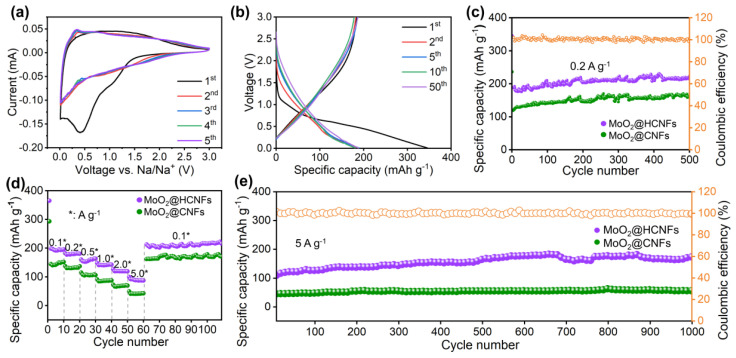
(**a**) CV curves of the MoO_2_@HCNF electrode at the scan rate of 0.2 mV s^−1^ for SIBs; (**b**) galvanostatic charge/discharge curves of the MoO_2_@HCNFs at 0.2 A g^−1^ for SIBs; (**c**) cycle performance, (**d**) rate performance, and (**e**) long cycle performance of the MoO_2_@HCNFs and MoO_2_@CNFs for SIBs.

The electrochemical measurements of the MoO_2_@HCNF and MoO_2_@CNF electrode materials were tested by assembling CR2025-type coin cells. Figure 4a displays the cyclic voltammetry (CV) curves of the MoO_2_@HCNFs in the voltage range from 0.01 to 3.0 V at a scan rate of 0.2 mV s^−1^. During the first curve, the reduction peaks were observed at 1.1 V and 0.4 V, corresponding to the formation of a solid electrolyte interphase (SEI) in the initial discharge cycle and the irreversible reactions [31,35]. There are no obvious oxidation peaks during the process of anodic scanning. After the activation of the first scan, a reduction peak located at 0.47 V and an oxidation peak located at 0.34 V were observed in subsequent scans, which are related to the conversion reaction between MoO_2_ and Mo. Furthermore, the CV curves were highly overlapped after the second cycle, showing high cycling stability and reflecting the strong reversibility of the composite electrode’s charging and discharging. The composite electrode shows excellent electrochemical activity, which proves the effectiveness and importance of the carbon nanofiber conductive network [28,31,36]. Figure 4b displays the charging and discharging curves of the hollow composite anode material for the 1st, 2nd, 10th, 50th, and 100th cycles. During the first discharge, a long voltage plateau started to appear at around 1.20 V, which corresponds to a reduction peak in the CV curve and finally reaches a high specific capacity of 347.14 mAh g^−1^. This voltage plateau disappeared in the subsequent cycles, and the specific discharge capacity decreased, representing the irreversible decomposition of the electrolyte and forming SEI. The later charge–discharge curves almost overlapped, indicating that the conversion reaction was highly reversible and MoO_2_ obtained good electrochemical stability in the composite electrode. Further, the cycling performance of the MoO_2_@HCNF and MoO_2_@CNF electrodes were investigated at 0.2 A g^−1^. The MoO_2_@HCNF electrode delivered excellent cycling stability with a reversible specific capacity of 221.55 mAh g^−1^ with 63.82% initial coulombic efficiency (ICE) after 500 cycles (Figure 4c). Notably, the MoO_2_@CNF electrode showed an inferior reversible specific capacity of 163.90 mAh g^−1^ after 500 cycles. To further explore the electrochemical performance of the MoO_2_@HCNF electrode, the rate capability is shown in Figure 4d. The MoO_2_@HCNF (or MoO_2_@CNF) electrode delivered high discharge capacities of 195.55 (155.49), 180.98 (135.20), 163.81 (109.71), 144.05 (90.46), 121.16 (71.21) and 88.90 (44.68) mAh g^−1^ at current densities of 0.1, 0.2, 0.5, 1.0, 2.0 and 5.0 A g^−1^, respectively. This could be restored to 222.08 (171.09) mAh g^−1^ when the current density was restored to 0.1 A g^−1^, which indicates that the MoO_2_@HCNF electrode has excellent multiplication performance. To further investigate the cycling stability, the MoO_2_@HCNF electrode was tested at a high current density of 5 A g^−1^ after ultra-long cycling (Figure 4e). After 1000 cycles, the discharge capacity of MoO_2_@HCNF electrodes increased from the initial 110.46 mAh g^−1^ to 174.10 mAh g^−1^, which could be attributed to the continuous activation of the electrode materials during the cycling process. At the beginning of the cycle, the permeability of the electrolyte is not good, and Na^+^ cannot diffuse into the electrode material quickly, so the sodium storage capacity is low at the beginning of the cycle. As the cycle proceeds, the electrolyte gradually penetrates into the interior of the electrode material, and the advantage of the utilization of the hollow structure increases significantly, so the specific capacity increases gradually. In contrast, the discharge capacity of the MoO_2_@CNF electrode was poorer, with an initial capacity of 47.42 mAh g^−1^ at a high current density and 57.02 mAh g^−1^ after 1000 cycles, which was much lower than that of the MoO_2_@HCNF electrode. The higher capacity of the MoO_2_@HCNF electrode compared with the MoO_2_@CNF electrode can be attributed to the more active sites provided by the hollow structure, which is favorable for the contact and reaction between MoO_2_ and electrolyte in the electrode. At the same time, the buffer space provided by the hollow carbon wall can work with the carbon layer to buffer the volume expansion of MoO_2_ and maintain the structural stability and cycling stability of the composite.

**Figure 5:**
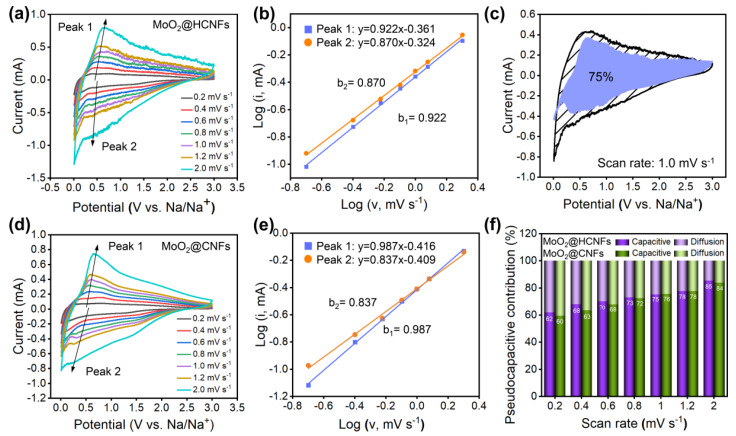
(**a**) CV curves of the MoO_2_@HCNFs at different scan rates; (**b**) corresponding log(i) versus log(ν) linear relationships at peaks; (**c**) pseudocapacitive contribution of the MoO_2_@HCNFs at scan rates of 1.0 mV s^−1^; (**d**) CV curves of MoO_2_@CNFs at different scan rates; (**e**) corresponding log(i) versus log(ν) linear relationships at peaks; (**f**) pseudocapacitive contribution of the MoO_2_@HCNFs and MoO_2_@CNFs at different scan rates.

To investigate the reaction kinetics of the MoO_2_@HCNFs and MoO_2_@CNFs, CV tests were performed at different scan rates from 0.2 mV s^−1^ to 2.0 mV s^−1^. The position of the redox peaks was only slightly shifted when the scan rate increased from 0.2 mV s^−1^ to 2.0 mV s^−1^, which shows superior cycling stability and reversible performance (Figure 5a,d). The relationship between the peak current (i) and the scan rate (v) can be described by Equations (1) and (2): [36,37]

i = av^b^ (1)

log (i) = blog (v) + log (a)(2)

where a and b are variable constants. The b value is the slope of the plots of log (i) versus log (v). When the b value is close to 0.5, the electrochemical behavior is mainly a diffusion-controlled process, while the b value is approaching 1.0, which represents a capacitive-controlled process. The b values of MoO_2_@HCNFs (or MoO_2_@CNFs) were 0.870 (0.837) and 0.922 (0.987) for the cathodic and anodic peaks, respectively (Figure 5b,e). This confirms that the electrochemical behaviors of the MoO_2_@HCNFs and MoO_2_@CNFs are represented by the predominance of the surface capacitive controlled process. To further investigate the pseudocapacitive contribution, Equations (3) and (4) are given [38,39]:

i = k_1_v + k_2_v^1/2^ (3)

i/v^1/2^ = k_1_v^1/2^ + k_2_ (4)

where k_1_v is the surface capacitive behaviour and k_2_v^1/2^ is the diffusion-controlled behaviour. As shown in Figure 5c, the pseudocapacitance contribution of the MoO_2_@HCNFs is 75.0% at a scan rate of 1.0 mV s^−1^, indicating that the pseudocapacitance behavior accounts for a higher proportion of the overall capacitance. Furthermore, we calculated the pseudocapacitance contribution of the MoO_2_@HCNFs and MoO_2_@CNFs at different scan rates (Figure 5f). The surface capacitive proportion gradually increased as the scan rate increased, with values of 61.9%, 67.8%, 70.1%, 73.0%, 75.0%, 77.5%, and 85.4% at gradually increasing sweep speeds of 0.2, 0.4, 0.6, 0.8, 1.0, 1.2, and 2.0 mV s^−1^, respectively. In comparison, the MoO_2_@CNFs exhibit a capacitance share of 59.5%, 63.5%, 67.7%, 72.4%, 75.6%, 77.6%, and 83.9%, respectively. In addition, the MoO_2_@HCNFs have a higher pseudocapacitance contribution than MoO_2_@CNFs at scan rates of 0.2, 0.4, 0.6, 0.8, and 2.0 mV s^−1^. The higher pseudocapacitance contribution facilitates the fast transfer kinetics of Na^+^ during intercalation/extraction, thereby further enhancing the rate performance of the MoO_2_@HCNF electrode. Therefore, the MoO_2_@HCNFs exhibited stronger surface reaction dynamics based on calculations, showing that they possessed a higher pseudocapacitance contribution than the MoO_2_@CNFs. This suggests that the hollow channels constructed by the MoO_2_@HCNFs are crucial for the transport of sodium ions in the electrodes.

**Figure 6:**
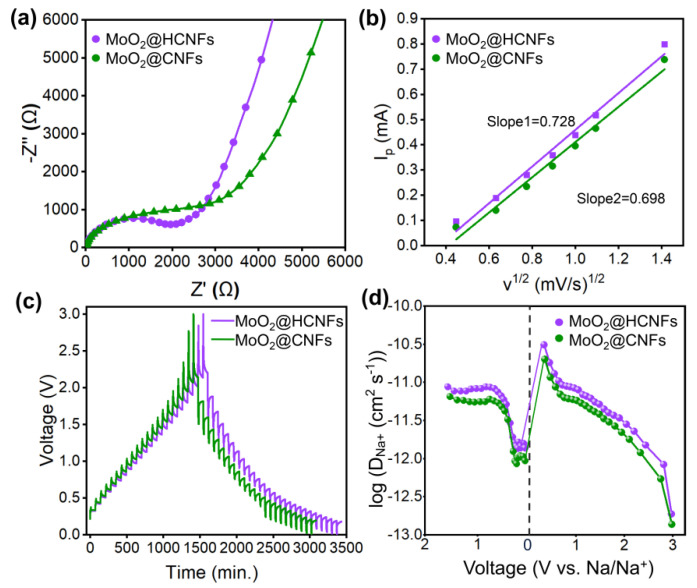
(**a**) Nyquist plots, (**b**) linear relationships between the peak current I_p_ and v^1/2^ of MoO_2_@HCNFs and MoO_2_@CNFs; (**c**) GITT tests; and (**d**) calculated sodium−ion diffusion coefficients of MoO_2_@HCNFs and MoO_2_@CNFs.

To further investigate the Na^+^ diffusion kinetics of the MoO_2_@HCNFs and MoO_2_@CNFs, electrochemical impedance spectroscopy (EIS) and the galvanostatic intermittent titration technique (GITT) were measured. The Nyquist plots shown in Figure 6a consist of a semicircle in the high-medium frequency region and a sloping line in the low-frequency region, with the semicircle region relating to the SEI film resistance (R_e_) and charge transfer resistance (R_ct_) and the sloping line corresponding to the Warburg impendence (Z_w_) of ion diffusion [39,40,41]. The MoO_2_@HCNF electrode exhibited a smaller R_ct_ than that of the MoO_2_@CNFs before cycling. This shows that the MoO_2_@HCNF electrode is capable of faster charge transfer and ion diffusion, demonstrating its excellent rate performance. Furthermore, the diffusion coefficient can also be calculated using the Randles–Sevcik equation [37,42]:

I_p_ = 2.69 × 10^5^An^3/2^CD^1/2^v^1/2^ (5)

where I_p_ corresponds to the peak current, A corresponds to the surface area of the electrode, n corresponds to the electron transfer number, C corresponds to the concentration of sodium ions, v corresponds to the scan rate, and D corresponds to the diffusion coefficient of sodium ions. As shown in Figure 6b, the slope of the line reflects the value of the Na^+^ diffusion coefficient. MoO_2_@HCNFs exhibits higher Na^+^ diffusion coefficients and is more beneficial to the transport of Na^+^ due to its steeper line. In addition, the diffusion behavior of Na^+^ is further investigated by the GITT curve, showing that the MoO_2_@HCNFs exhibited a longer time and higher diffusion coefficient of sodium ions during charging and discharging (Figure 6c,d). The Na^+^ diffusion coefficient (D_Na+_) could be calculated by Equation (6).

(6) $$D_{Na^{+}}=\frac{4}{\pi \tau }{\left[\left(\frac{m_{B}V_{m}}{AM_{B}}\right)\frac{\Delta E_{s}}{\Delta E_{t}}\right]}^{2}$$

where *τ* is the constant current titration time; *A* is the surface area of the electrode; *m_B_*_,_ *V_m_,* and *M_B_* are the mass, molar volume, and molar mass of active materials, respectively; and Δ*E_s_* and Δ*E_t_* are the total voltage change due to pulses and the voltage change for constant current charging/discharging, respectively. The diffusion coefficients of Na^+^ in MoO_2_@HCNFs calculated via GITT tests during discharging and charging were 8.74 × 10^−12^ to 1.37 × 10^−12^ cm^2^ s^−1^ and 3.09 × 10^−11^ to 1.87 × 10^−13^ cm^2^ s^−1^, while the diffusion coefficients of Na^+^ in the MoO_2_@CNFs were 6.49 × 10^−12^ to 9.30 × 10^−13^ cm^2^ s^−1^ and 2.01 × 10^−11^ to 1.36 × 10^−13^ cm^2^ s^−1^, respectively. These results suggest that hollow channels of the MoO_2_@HCNFs provide faster transport velocity of sodium ions, resulting in superior ion diffusion kinetics.

**Figure 7:**
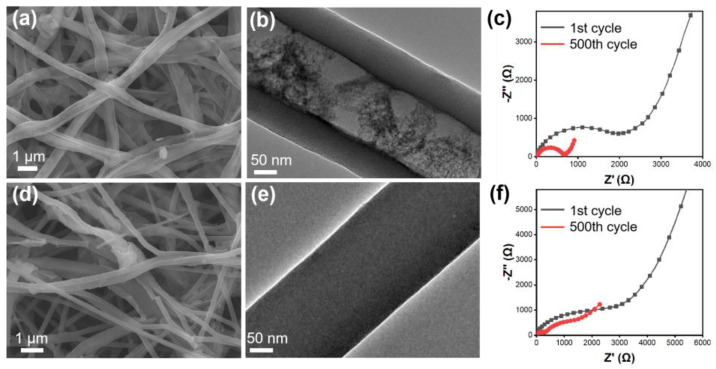
(**a**) SEM images, (**b**) TEM images, and (**c**) Nyquist plots of MoO_2_@HCNFs; (**d**) SEM images, (**e**) TEM images, and (**f**) Nyquist plots of MoO_2_@CNFs after 500 cycles at 0.2 A g^−1^ for SIBs.

The micromorphology and diffusion kinetics of the MoO_2_@HCNFs and MoO_2_@CNF electrodes were further investigated after 500 cycles at 0.2 A g^−1^ of SIBs. The integrity of the hollow carbon nanofiber structure in MoO_2_@HCNFs is remarkable, as it exhibited no signs of swelling or collapse after cycling. The overall morphology of the fibers closely resembled their pre-cycling state, with only minimal fiber breaks observed. Similarly, MoO_2_@CNFs experienced some fiber breaks and agglomerations between fibers (Figure 7a–e). The structure of the electrode material after 500 cycles of charging and discharging was not destroyed, indicating that the prepared hollow structure can provide a buffer space for the volume change in the electrochemical reaction process, and avoids the problem of structural collapse of the material during multiple charging and discharging cycles. This demonstrates the robustness of the hollow structure obtained from the preparation in this work, which is important for the long cycling performance of the electrode materials. In addition, the charge transfer resistance of both the MoO_2_@HCNFs and MoO_2_@CNFs decreased after 500 cycles, which is favorable for improving electrochemical performance (Figure 7c,f).

## 4. Conclusions

In summary, a high-performance free-standing MoO_2_@HCNF electrode was fabricated by means of facile coaxial electrospinning and subsequent heat treatment. It is revealed that the MoO_2_@HCNFs not only possessed a larger specific surface area and superior specific capacity but also delivered better rate capability and faster diffusion kinetics of Na^+^. The superior electrochemical performance may be related to the following advantages: (i) The larger specific surface area can provide more active sites, and MoO_2_ is uniformly loaded on the carbon nanofibers. At the same time, the 3D carbon network also increases the contact area between the electrode material and the electrolyte and provides more electronic channels. (ii) The ultra-long and excellent cycling performance is assigned to the hollow carbon nanofiber structure, which alleviates the volume change during charging and discharging while shortening the Na^+^ diffusion path and improving electron conductivity, leading to stronger ion transport and ultimately improved sodium storage performance. This work not only provides a simple and widely applicable strategy for the fabrication and utilization of electrode materials for high-performance energy storage batteries; the properties of the flexible film are also conducive to research on smart wearable devices.

## Data Availability

The data that support the findings of this study are available in the Supplementary Materials of this article.

## Supplementary Materials

The following supporting information can be downloaded at: https://www.mdpi.com/article/10.3390/polym16111452/s1, Figure S1. SEM images of (a) as−spun MoO_2_@CNFs and (b) MoO_2_@CNFs; (c) flexibility test of MoO_2_@HCNFs film; Figure S2. TGA curves of MoO_2_@HCNFs and MoO_2_@CNFs; Figure S3. (a) Survey XPS spectra of the MoO_2_@HCNFs and (b) high-resolution XPS spectra of O 1s; Table S1. Comparison of sodium storage performance of MoO_2_−based anode materials [43,44,45].
