# Supplementary material for: Stable Structure and Fast Ion Diffusion: A Flexible MoO2@Carbon Hollow Nanofiber Film as a Binder-Free Anode for Sodium-Ion Batteries with Superior Kinetics and Excellent Rate Capability
Source: Polymers (Basel). 2024 May 21;16(11):1452. doi: 10.3390/polym16111452 (PMC11174558; doi:10.3390/polym16111452)
Supplement: Supplementary file 1 [file polymers-16-01452-s001.zip › polymers-2985163-supplementary.pdf]

polymers

Supporting Information

Stable Structure and Fast Ion Diffusion: A Flexible MoO_2@Carbon Hollow Nanofiber Film as a Binder-free Anode for Sodium-ion Batteries with Superior Kinetics and Excellent Rate Capability

Na Feng, Mingzhen Gao, Junyu Zhong, Chuantao Gu, Yuanming Zhang * and Bing Liu *

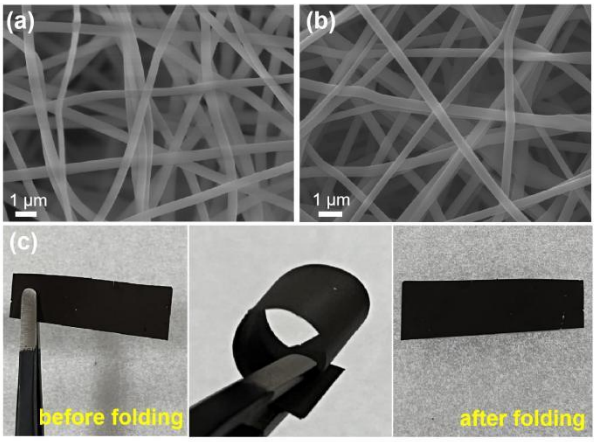

Figure S1. SEM images of (a) as−spun MoO_2@CNFs and (b) MoO_2@CNFs; (c) flexibility test of MoO_2@HCNFs film.

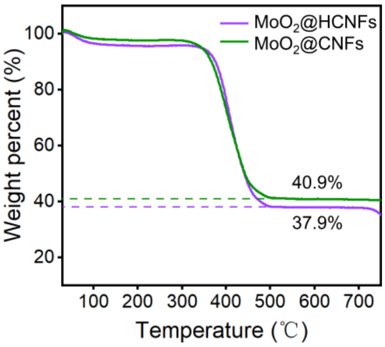

Figure S2. TGA curves of MoO_2@HCNFs and MoO_2@CNFs.

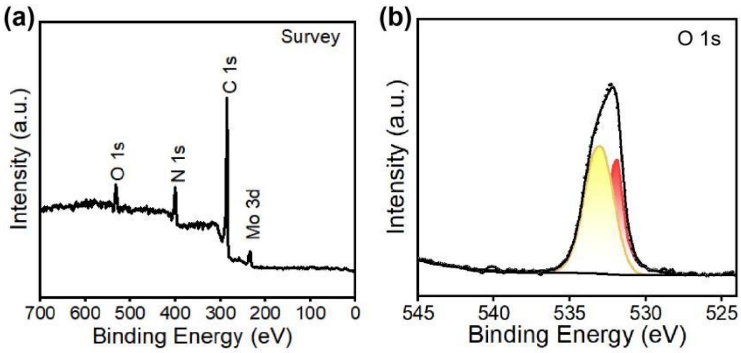

Figure S3. (a) Survey XPS spectra of the MoO_2@HCNFs and (b) High−resolution XPS spectra of O 1s.

Table S1. Comparison of sodium storage performance of MoO_2–based anode materials.

Materials	Capacity/current density/cycle number [mAh g^{-1}/A g^{-1}/–]	Diffusion coefficients of Na^+ (D_{Na+}) [cm^2 s^{-1}]	References
MoO_2/GO	345/0.1/100; 276/0.1/1000	-	35
MoO_2/C nanocomposite	165.4/1C/100; 95.0/2C/100	-	43
S-doped MoO_2/C nanofibers	160/1/600; 86/5/1200	-	30
MoO_2	94.7/0.2/5; 47.8/3/300	-	44
TiO_2@MoO_2-C	210/1/500; 110/5/10000	-	36
MoO_2/C	367.8/0.05/100	-	28
MoO_2@N-doped C nanofibers	350/0.1/200	-	24
MoO_2@MoS_2/rGO	496.8/0.1/100; 362.5/1/300	-	31
MoO_2@CNF	223.6/0.1/100; 152.9/1/1000	~$10^{-9.5}$ to ~$10^{-8.5}$	45
TiO_2@MoO_2	233/0.2/500; 175/1/1000	~10^{-10} to ~10^{-9}	38
MoO_2@HCNF	221.55/0.2/500; 174.10/5/1000	8.74×10^{-12} to 1.37×10^{-12}	This work
